# Harvesting pre-polarized macrophages using thermo-responsive substrates

**DOI:** 10.1038/srep42495

**Published:** 2017-02-14

**Authors:** Vera Malheiro, Yvonne Elbs-Glatz, Magdalena Obarzanek-Fojt, Katharina Maniura-Weber, Arie Bruinink

**Affiliations:** 1 Laboratory for Biointerfaces, Empa, Swiss Federal Laboratories for Materials Science and Technology, Lerchenfeldstr. 5, CH-9014 St. Gallen, Switzerland

## Abstract

In the cell culture environment macrophages are highly adherent cells. Currently used methods to harvest macrophages have the disadvantage of reducing cell viability and their ability to re-attach after seeding. Although thermo-responsive surfaces have been employed to harvest cell sheets no reports are available to use these to harvest (pre-polarized) macrophages. We show that this method significantly improves the yield of living macrophages and percentage of subsequent cell reattachment, whilst having a minimal effect on the cell phenotype.

Monocytes/macrophages play a key role in immune related processes and tissue homeostasis^1^,^2^. In addition, it became clear that macrophages also have important regulatory functions regarding tissue regeneration and implant integration. Macrophages can exhibit various states of cell polarization in addition to their resting state (called M(−) or naïve)^3^. Currently, the most frequently described functional states of polarization are the M1-like inflammatory and M2-like regenerative phenotypes (and their activation condition dependent sub-states)^3^.

In order to polarize resting state macrophage towards M1-like and M2-like macrophages polarizing agents are added to the culture medium. For instance, to obtain M1-like macrophages lipopolysaccharides (LPS) are generally introduced. However, LPS not only polarize macrophages but also will adsorb to the substratum and by that contaminate it^4^. In case cells are subsequently used without harvesting, for instance to investigate the effects of the substratum or of added compounds on the macrophage phenotype, the latter contamination may induce artefacts. This contamination possibly falsifies the experimental outcome, since it may interfere with the interaction between the macrophage and the biomaterial or affect the cells directly even if medium is replaced by LPS-free medium^4^,^5^,^6^. By harvesting macrophages and seeding them afterwards using medium without polarizing agents the latter can be circumvented.

One characteristic of cultured macrophages, especially of activated ones, is their very strong adhesion to any substratum^7^,^8^,^9^ and as a consequence they are difficult to harvest. Commonly used methods to harvest macrophages cultivated on tissue culture treated polystyrene (TCPS) surfaces are mechanical treatments such as scraping^10^, treatment with the calcium chelating agent ethylene-diamine-tetra acetic acid (EDTA) with or without subsequent scraping^11^,^12^,^13^, enzymatic treatment e.g. trypsin with or without subsequent scraping^14^,^15^ or trypsin treatment combined with EDTA^16^. However, due to the strong adhesion to the substratum the cell harvesting yield is rather low using these classical methods. The harvesting methodology may affect cell viability of the harvested cells, and also influence and change the functional state of this highly responsive cell type^17^. For instance, it is reported that a trypsin treatment may greatly influence the cell membrane protein composition, reactivity (at least transiently) of the cell to medium components and cell membrane permeability^18^,^19^,^20^. Additionally, one limiting factor for the experimental use of harvested pre-polarized macrophages could be the phenotypic stability of the cells, i.e. the stress evoked by cell harvesting and reseeding may affect this phenotype stability^17^. Already without harvesting it is known that M1- and M2-like macrophages regain the resting state phenotype if medium is replaced by and cultivated for in medium without polarizing agents^21^. They found, after cultivation for 6 days in cytokine-free medium, the percentages of CD64^+^, CD80^−^ M1-like cells or CD11b^+^, CD209^−^ M2-like cells were reduced by at least 50% with concomitant increases in the fraction of CD64^+^, CD80^−^ or CD11b^+^, CD209^−^ M(−) cells. These cells fully reverted back to M(−) state by day 12.

More than two decades ago, Yamada and co-workers introduced a new enzyme-free method to recover cell-sheets using a TCPS substrate coated with the thermo-responsive poly(N-isopropylacryl-amide) (pNIPAm)^22^. Below the transition temperature of the polymer, the conformation of thermo-responsive brushes changes and the surface becomes protein repellent. As a consequence, the cells that are bound to the surface through these proteins are released from the surface.

Here we report that the use of commercially available pNIPAm based thermo-responsive surfaces to culture, polarize and harvest macrophages is superior to the commonly used method, i.e. cultivation on TCPS and harvesting using EDTA plus scraping. The selection of EDTA plus scraping as reference harvesting procedure was based on literature^23^,^24^ and on our own experience; in comparison to enzyme based methods it gave the highest cell yield.

A serial experimental procedure was defined to prove the superiority of the pNIPAm based methodology (Fig. 1) and to answer the following 5 questions. (1) Does cultivation on pNIPAm affect monocyte activation towards M(−) macrophages? (2) Is the polarization potential of macrophages on pNIPAm altered in comparison to cells on TCPS? (3) Has pNIPAm an advantage over TCPS based harvesting in terms of yield of harvesting and cell viability of harvested M(−), M1-like and M2-like cells? (4) After reseeding of cells on TCPS, do macrophages harvested from pNIPAm have an advantage over those harvested from TCPS in terms of efficiency to reattach after cell seeding, and (5) is there a difference in M(−), M1-like and M2-like phenotypic stability after reseeding of cells on TCPS? To answer these questions the human THP-1 monocytic leukemia cell line have been selected as a model based on the fact that its cellular processes are well-characterized and since it is one of most widely used cell lines to investigate the function and regulation of monocytes and macrophages^25^,^26^. Using our experimental set-up we obtained the following results:

## Activation of THP-1 cells towards adherent M(−) resting state

In contrast to primary cells, monocytes from cell lines, like the human THP-1 used in this study, require stimulation for maturation to the M(−) state. Phorbol-12-myristate-13-acetate (PMA) was used for this induction. To prove that the sensitivity to PMA was not affected by the substratum THP-1 cells were cultured on TCPS and pNIPAm substrates in medium containing PMA at the concentrations 25, 50, 100 and 300 nM for 3 days. The evaluated PMA concentrations did not differently affect the number of adhered cells if cultured on TCPS and pNIPAm, as determined by DNA amount (Fig. 2). Additionally, light microscopic examination did not reveal any differences between cells cultured on TCPS and pNIPAm in the presence of different PMA concentrations. The commonly used concentration of 100 nM PMA was selected for the subsequent study.

## Macrophage polarization

**Figure 1:**
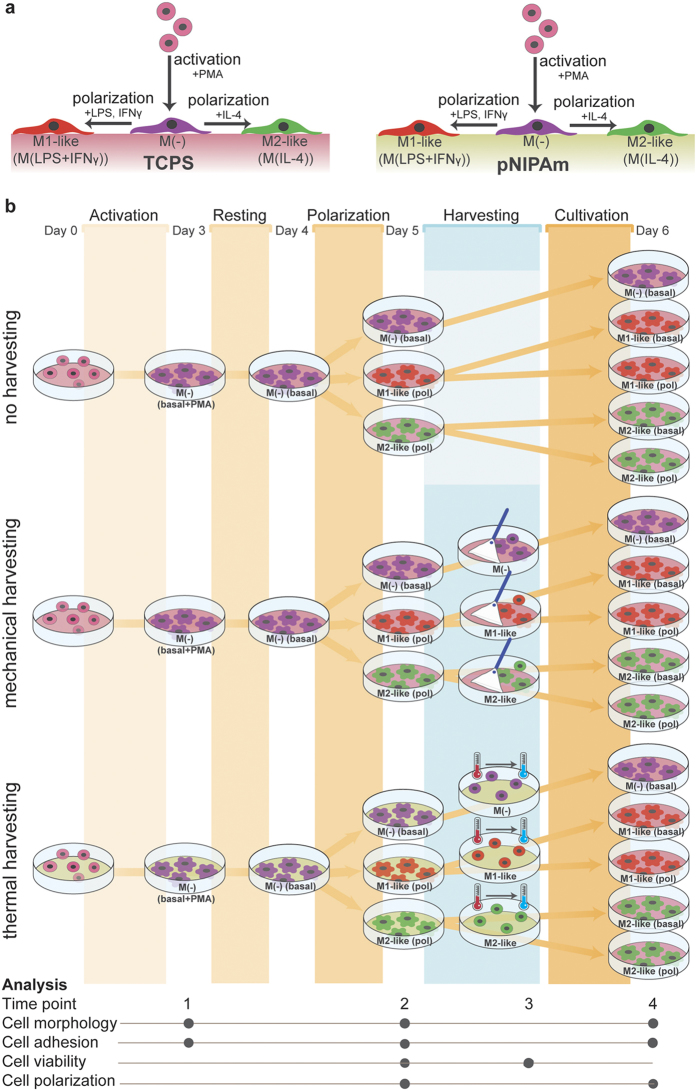
Scheme of the experimental set-up indicating the method of differentiation of THP-1 monocytic cells towards the investigated M(−), M1- and M2-like macrophages (**a**) and the time sequence plus time points of analysis (**b**). Basal: cells in basal medium; pol: cells in polarizing medium containing polarizing compounds.

**Figure 2:**
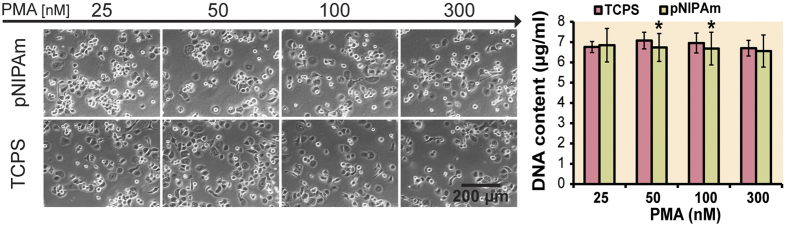
THP-1 cell cultures on TCPS and pNIPAm plates after treatment with 25–300 nM PMA for 3 days (time point 1). (**a**) Light microscopy pictures of the cultures before removal of non-adherent cells. (**b**) Total culture DNA content after removal of non-adhered cells. Data obtained from 4 independent experiments are presented as mean ± sdev. *p < 0.05 different from cultures on TCPS.

**Figure 3:**
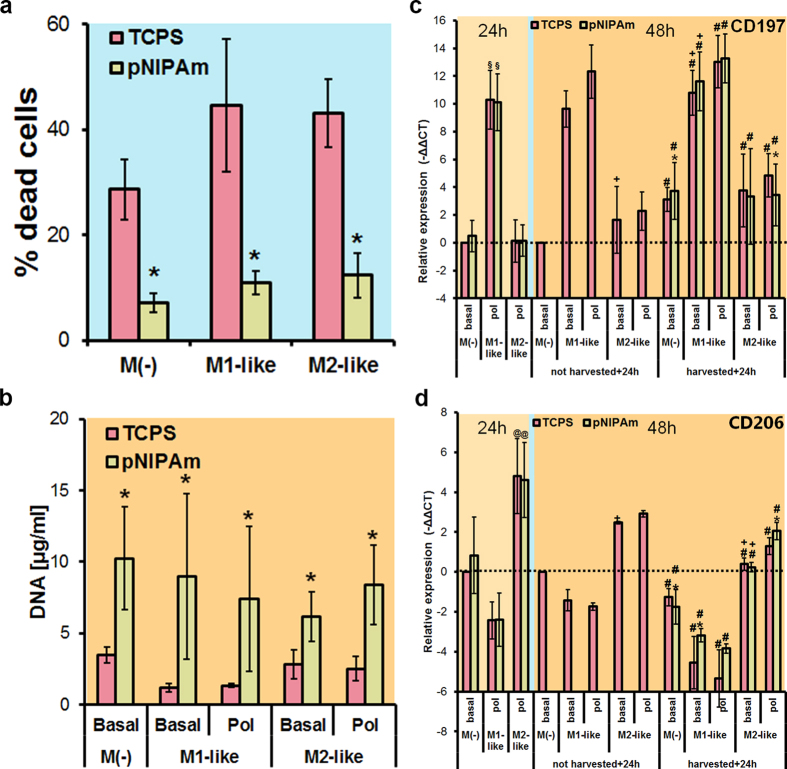
Comparison of cells cultured on TCPS and pNIPAm plates and subsequent isolation. (**a**) Effect of harvesting technique (cells kept on TCPS and harvested using EDTA treatment plus scraping, versus cells kept on pNIPAm and harvested by cooling down the cultures) on the percentage of dead cells in the isolates determined by flow cytometry analysis of cells stained with ethidium homodimer. Data from 3 independent experiments are presented as mean ± sdev. *Significantly different from TCPS (p < 0.05). (**b**) Effect of harvesting technique on the number of attached cells, determined by the quantification of DNA 24 h after reseeding. Data from 4 independent experiments are presented as mean ± sdev. *Significant difference between pNIPAm and TCPS in paired comparison (p < 0.05). (**c**) Effects of surfaces and harvesting technique on THP-1 polarisation relative to resting state cells (M(−)) on TCPS of the same dataset taking quantity of CD197 (A) and CD206 (B) mRNA relative to GAPDH as index. Data of TNFα, CCL22, IL-10, and CXCL10 as well as full data of CD206 and CD197 are shown in Supplementary Fig. 2. Dashed lines represent the control level, i.e. 0 being the −ΔΔCt of M(−) cultures on TCPS. Data are presented as mean ± sdev over 3 independent experiments. *significantly different from identically treated cultures but cultured on TCPS surface. §significantly different from M(−) state cultured on TCPS of the same culture time and dataset. ^+^Significant difference in values comparing identically treated cultures that in the period 24 to 48 h after start of the polarization step are kept in polarisation medium (pol) instead of in basal medium (basal) ^#^significantly different from not harvested, identically treated cultures on TCPS (p < 0.05).

After 3 days of treatment with 100 nM PMA followed by one day in PMA-free basal medium, cells on both types of substrate were either incubated in medium with LPS (100 ng/mL) plus interferon-γ (IFγ)(20 ng/mL) to induce a M1-like phenotype or with IL-4 (20 ng/mL) to obtain a M2-like phenotype (Fig. 1a)^3^,^27^,^28^,^29^,^30^. Cells cultivated in basal medium were assumed to express the resting state M(−) macrophage-like phenotype. Macrophages polarized into M1-like or M2-like cells on both TCPS and pNIPAm as indicated by gene expression and cytokine release as assessed after 6 and 24 h, respectively 24 h after start of treatment (Fig. 3, Supplementary Figs 2 and 3). As expected, M1-like polarized macrophage cultures were characterized by high expression levels of pro-inflammatory markers TNF-α, CXCL10, and CD197 mRNA as well as TNF-α release, and M2-like polarized macrophage cultures by expression of CD206 and CCL22 mRNA. After 6 h gene expression of IL-10 and after 6 and 24 h the concentration of IL-10 protein in the medium was increased in M1-like polarized cells. No clear difference was observed for IL-10 mRNA expression between the M1-like and M2-like polarized cells after 24 h. IL-10 has been reported as a marker for some M2-like sub-states of macrophages but also for M1-like cells^3^,^31^,^32^. Besides this, also the quantity of LDH, that was released by cells cultivated on TCPS and pNIPAm, was comparable as assessed 24 h after addition of polarising agents (Supplementary Fig. 4). Furthermore, the number of adhered cells on TCPS and pNIPAm was found to be the same taking the amount of total DNA on the culture plate as an index (Supplementary Fig. 5). Thus no evidence was found that TCPS and pNIPAM substrata differently affect THP-1 cells. This is in line with findings of Fan and co-workers who reported that unstimulated RAW264.7 macrophage-like cells exhibit similar characteristics (DNA amount of attached cells, proliferation rate, IL-1β release, surface expression of CD80 and MHC-II) on TCPS as seen on solvent casted pNIPAm surfaces as measured after 48 h of cultivation^19^.

## Cell harvesting

Large differences between cells cultured on TCPS and pNIPAm became apparent during and after cell harvesting. By using the pNIPAm based lift-off technique, the percentage of dead cells post-harvesting was reduced by around 75% in comparison to the EDTA/scraping technique (Fig. 3a; Supplementary Fig. 6). Furthermore, less time was needed to harvest macrophages by the pNIPAm based thermal lift-off technique when compared to EDTA and scraping as cell harvesting method.

## Cell attachment to TCPS after harvesting

After harvesting, M(−), M1- and M2-like cells from TCPS and pNIPAm plates were reseeded on TCPS using for all conditions identical number of viable cells. Light microscopy analysis of the cell cultures 24 hours after reseeding revealed poor attachment of cells that were harvested with the EDTA/scraping method in comparison to cells harvested by the pNIPAm temperature lift-off approach (Supplementary Fig. 7). This was further confirmed by quantifying the amount of total DNA from attached cells 24 h after seeding (Fig. 3b). The number of attached cells, which were previously harvested from pNIPAm plates, was at least two times higher than that of cells collected from TCPS by EDTA/scraping procedure. Our data indicate that not only fewer living cells are obtained with this commonly used method, but also that the chemical (EDTA) plus mechanical (scraping) stress significantly reduces the cell potential to re-attach to TCPS after harvesting. It may be noted that in our hands this cell yield was even lower using Trypsin/EDTA as cell harvesting method if compared with the EDTA plus scraping method (data not shown).

## Macrophage state of polarization after harvesting and reseeding

Cells obtained from pNIPAm plates expressed similar levels of CXCL10, CD197 and CD206. However, higher levels of CCL22, TNF-α and IL-10 mRNA were found in case cells were obtained from pNIPAm surfaces (Fig. 3c,d; Supplementary Figs 2 and 3). Another key aspect is the phenotypic stability of this highly responsive cell type^21^. This stability might be (differently) modified based on the harvesting procedure. The phenotypic stability was assessed by comparing M1-like and M2-like specific gene expression after reseeding of cell harvested from TCPS and pNIPAm and subsequent cultivation in medium either without or with polarizing components. No clear-cut differences were found in gene expression comparing identically treated cultures but being differently harvested.

It may, however, be noted that regardless of the substratum and initial polarization protocol the extent of polarization was highly reliant on the continuous presence of polarization agents. We observed that in case of M1-like polarized cells, the omission of LPS and IFγ in the reseeding medium resulted in a significant reduction in the expression of M1-like (but not of the M2-like) cell markers. Similarly, in case of M2-like polarized cells the omission of IL-4 resulted in a significant reduction in the expression of M2-like (but not of the M1-like) cell markers. Consequently, there are clear time boundaries for investigations using pre-polarized cells in absence of polarizing agents.

The benefits of using thermo-responsive surfaces to harvest resting state and polarized macrophages can be summarized as follows: i) monocytes can be activated towards resting state macrophages and polarized towards the M1- and M2-like functional states nearly identical to those cultivated on TCPS; ii) the methodology enables a very gentle cell isolation without compromising cell viability, which in contrast to harvesting from TCPS; iii) the ability of the living macrophages harvested from pNIPAm to reattach to the surface of interest is significantly increased; iv) cells obtained from pNIPAm and reseeded on TCPS exhibit similar characteristics as to those that were not harvested; v) the phenotypic stability is not affected by the harvesting procedure, and vi) the present methodology enables the investigation of the interactions between pre-polarized macrophages and biomaterial surfaces in absence of polarizing agents.

## Methods

### Cell culture

The human monocytic cell line THP-1 (ECACC, UK) was cultured in basal medium (RPMI1640 supplemented with 10% foetal calf serum, 2 mM L-glutamine (final concentration) (Sigma Aldrich, CH) and 1% 100X antibiotics (PSN; Life Technology, CH)) and sub-cultured before reaching 1.0 × 10^6^ cells/mL. The general procedure was that THP-1 were seeded at a density of 1.1 × 10^5^ living cells/cm^2^ onto tissue culture treated polystyrene (TCPS) cell culture dishes (TPP, Bioswisstec, CH) and (poly(N-isoproplyacrylamide)-grafted polystyrene dishes (pNIPAm) produced by electron beam irradiation, Nunc UpCell plates^33^,^34^, Thermo Fisher Scientific-Nunc A/S, Denmark) plates in basal medium containing 25–300 nM phorbol 12-myristate 13-acetate- (PMA) (Sigma; CH) in order to differentiate the cells towards resting state (M(−)) macrophages. After incubation for 3 days, cells were washed with basal medium and cultured for another 24 h in basal medium without additions. Subsequently, medium was replaced by fresh basal medium with either no additions for keeping cells in the M(−) state, with components inducing a cell polarization, i.e. lipopolysaccharide (LPS; Sigma; CH) (100 ng/mL) combined with interferon-γ (20 ng/mL) (Miltenyi Biotec, D) to induce M(LPS+ IFNγ) being one representative of the M1-like states or interleukin (IL)-4 (20 ng/mL) (Miltenyi Biotec, D) to polarize cells towards M(IL-4) being one representative of the M2-like states^3^. In the present study we will term these cells as M1- and M2-like being a currently commonly used designation (e.g. refs 35 and 36). After 24 h, cultures were washed with basal medium. Thereafter, pNIPAm plates (with basal medium on top) were cooled down in the fridge for 20–40 min to release cells from the plate. Remaining attached cells were suspended by a gentle repeated pipetting. In case macrophages were cultured on TCPS plates cells were harvested according^37^,^38^. For this cultures were washed with 37 °C warm PBS (without Mg^2+^ and Ca^2+^) cultures. Thereafter, cold PBS without Mg^2+^ and Ca^2+^ containing 10 mM EDTA (Sigma-Aldrich, CH) was added to the cultures and incubated for 10–15 min at 4 °C and subsequently scraped off with a cell scraper. A decrease in temperature from 37 °C to 4 °C is known to reduce cell-substratum adhesion strength^39^. The isolated cells were washed directly afterwards using basal medium. After a centrifugation step to remove the dead cells, cells were reseeded onto 6-well TCPS plates at a density of 1.1 × 10^5^ living cells/cm^2^ in basal medium or the corresponding polarization medium. The obtained cultures were cultivated for 24 h.

In order to compare the cell performance on pNIPAm and TCPS plates and to assess the effect of the harvesting procedure, cultures were analysed at 4 different time points (Fig. 1):

- *Time point 1* (after PMA treatment): Light microscopy pictures of the cultures were acquired. Cultures were washed with PBS and total DNA was assessed using the Hoechst 33342 assay.
- *Time point 2* (after cell polarization): TNF-α and IL-10 concentrations in the culture medium were determined by enzyme-linked immunosorbent assay (ELISA) and LDH release was assessed. Of a second group of cultures, total DNA was determined after a wash with warm PBS and of a third group mRNA was isolated for assessing gene expression (6 and 24 h after onset of polarization).
- *Time point 3* (just after harvesting): The percentage of dead cells was determined by trypan blue exclusion test and by flow cytometry quantifying the number of cell able to take up ethidium homodimer.
- *Time point 4* (24 h after re-seeding on TCPS): Light microscopy pictures were taken and medium concentration of TNF-α and IL-10 were determined by ELISA. After washing the cultures with PBS, total culture DNA was quantified using the Hoechst 33342 assay. Of the remaining cultures mRNA was isolated for assessing gene expression.

### RNA extraction and purification

RNA from adherent cells was isolated by the spin column method using a commercially available RNeasy Mini Kit (QIAGEN^®^, D), according to the manufacturer’s instructions. The RNA concentration and quality were determined using a Nanodrop ND-1000 Spectrophotometer (Thermo Fisher Scientific, CH). Only RNA with an optical density (OD) 260/280 ratio between 1.9 and 2.1 was used for PCR analysis.

### Real-time reverse transcription–polymerase chain reaction

Complementary DNA (cDNA) was synthesized from 400 ng of total RNA using the iScript cDNA Synthesis Kit (Bio-Rad Laboratories) in a total reaction volume of 20 μL using a CFX96^™^ Real-Time PCR Detection System (Biorad Laboratories). A temperature profile of 5 min priming at 25 °C, followed by the reverse transcription at 42 °C for 30 min and the reverse transcription inactivation at 85 °C for 5 min was performed. After a final cool down to 4 °C, the cDNA was diluted 1:5 in RNAse free water and stored at −80 °C for subsequent use.

Primer pairs - designed over exon–exon junctions using PrimerBlast online software (http://ncbi.nlm.nih.gov/tools/primer-blast/) and manufactured by Microsynth AG (Balgach, CH) – are displayed in Supplementary Table S1. Primer pairs were evaluated for the efficiency of amplification and only pairs with efficiency between 90% and 110% were used in this study.

Quantitative real-time PCR was performed using 5 μL of cDNA sample and 7 μL of 0.2 μM forward and reverse primer of which 6 μL iQ SYBR Green Supermix (Biorad Laboratories) using a CFX96^™^ Real-Time PCR Detection System (Biorad Laboratories). The cycling conditions were as follows: an initial 95 °C for 3 min, followed by 40 cycles of 95 °C for 10 s and 57 °C for 30 s. Then, a melting curve was monitored by heating from 65 °C to 95 °C in temperature steps of 0.5 °C.

Fold changes in gene expression were taken using the −ΔΔCt method^40^; each gene was normalized against GAPDH. In the illustrations M(−) cultivated on TCPS were designated as the calibrator.

### TNF-α and IL-10 secretion from THP-1 cells

Supernatants of THP-1 cultures stimulated as indicated above were collected, centrifuged (250 RCF, 4 °C) and stored at −80 °C until assaying. Concentration of TNF-α and IL-10 in culture supernatants was determined by ELISA (Human IL-10 or TNF-α ELISA Ready-SET-Go!^®^, eBioscience) according to manufacturer’s protocol. For the detection of TNF-α, cell culture supernatants were pre-diluted (1:25) in complete medium to achieve a concentration within the range of the standard curve. Sample concentrations (pg/mL) were determined by measuring the mean absorbance values with an ELX800 Bio-Tek microplate reader at 630 nm wavelength, and converting the signal to cytokine concentration using a standard calibration curve.

### Lactate dehydrogenase activity (LDH) and uptake of ethidium homodimer

LDH activity was determined in the cell culture supernatant in comparison to cultures treated with a lysis buffer (Promega CytoTox96 Non-Radioaktive Cytotoxicity Assay, USA).

The percentage of dead cells was determined after harvesting. For this, the obtained cell suspension was treated with ethidium homodimer (ThermoFisher Scientific, CH) for 10 min at a final concentration of 8 μM to label the dead cells. Thereafter, the percentage of fluorescent cells was quantified by flow cytometry using unlabelled and labelled 0.2% digitonin permeabilized cell suspensions as references (GALLIOS, BeckmanCoulter International S.A., CH). The percentage of dead cells was defined by the ratio between number cells exhibiting ethidium homodimer fluorescence intensity above threshold and the total number of cells. Examples of the measurements are presented in Supplementary Fig. 1. Additionally, number of dead cells was determined using the trypan-blue exclusion test.

### Statistics

Data are presented as mean ± standard error of the mean (SE) from a minimum of three independent experiments. In case of mRNA, data was analysed for statistical significance employing the TIBCO Spotfire S ^®^ Plus version 8.1 software using post-hoc two-way multiple comparison ANOVA (analysis of variance) Bonferroni test to detect differences in the fold change levels using the ΔCt values (gene of interest minus GAPDH Ct value) (2 identical treated cultures per experiment with 3 independent experiments in total; time point 2 and 4). The same test was used to compare DNA values (3 identical treated cultures per experiment with 4 independent experiments in total; time point 1) (2 identical treated cultures per experiment with 4 independent experiments in total; time point 4) and LDH values (2 identical treated cultures per experiment with 3 independent experiments in total; time point 3).

The paired student t-test was used to compare DNA values (1 identical treated culture per experiment with 3 independent experiments in total; time point 2), to detect significant differences in percentage of dead cells of cultures harvested from TCPS and pNIPAm plates (1 identical treated culture per experiment with 3 independent experiments in total; time point 3) and to determine significant differences in IL-10 and TNF-α release in cultures kept on TCPS and pNIPAm (in each of the 3 independent experiments the mean cytokine values in the supernatant of 3 identical treated cultures was normalised to the DNA values obtained from 1 (time point 2) or 2 (time point 4) identically treated cultures of which the non-adherent cells were removed before measurement). Confidence level was set to 0.95.

## Additional Information

**How to cite this article**: Malheiro, V. *et al*. Harvesting pre-polarized macrophages using thermo-responsive substrates. *Sci. Rep.* **7**, 42495; doi: 10.1038/srep42495 (2017).

**Publisher's note:** Springer Nature remains neutral with regard to jurisdictional claims in published maps and institutional affiliations.

## Supplementary Material

Supplementary Information
