# Supplementary material for: Harvesting pre-polarized macrophages using thermo-responsive substrates
Source: Sci Rep. 2017 Feb 14;7:42495. doi: 10.1038/srep42495 (PMC5307341; doi:10.1038/srep42495)
Supplement: Supplementary Information [file srep42495-s1.pdf]

Article

Harvesting pre-polarized macrophages using thermo-responsive substrates

Vera Malheiro, Yvonne Elbs-Glatz, Magdalena Obarzanek-Fojt, Katharina Maniura-Weber, Arie Bruinink

Laboratory for Biointerfaces, Empa, Swiss Federal Laboratories for Materials Science and Technology, Lerchenfeldstr. 5, CH-9014 St. Gallen, Switzerland

Supplementary information Tables and figures

Supplementary table 1 ¦ List of primer pairs used for gene expression profiling

Gene	Abbreviation	PCR primers (5'-3')	Product length (bp)
Tumor necrosis factor alpha	TNF-a	Fw. CCGTCTCCTACCAGACCAAG<br>Rv. CTGAGTCGGTCACCCTTCTC	148
Interleukin 10	IL-10	Fw. ACATCAAGGCGCATGTGAAC<br>Rv. CAGGGAAGAAATCGATGACAGC	85
C-X-C motif chemokine 10	CXCL10	Fw. CAGTCTCAGCACCATGAATCAA<br>Rv. CAGTTCTAGAGAGAGGTACTCCTTG	95
C-C motif chemokine 22	CCL22	Fw. GCGTGGTGTTGCTAACCTTC<br>Rv. CCACGGTCATCAGAGTAGGC	115
C-C chemokine receptor type 7	CD197	Fw. GTGGTTTTACCGCCCAGAGA<br>Rv. CACTGTGGTGTTGTCTCCGA	142
Mannose receptor C type 1	CD206	Fw. GCTACCCCTGCTCCTGGTTT<br>Rv. CGCAGCGCTTGTGATCTTCA	101
Glyceraldehyde-3-phoshate dehydrogenase	GAPDH	Fw. AGTCAGCCGCATCTTCTTTT<br>Rv. CCAATACGACCAAATCCGTTG	97

Supplementary Figures

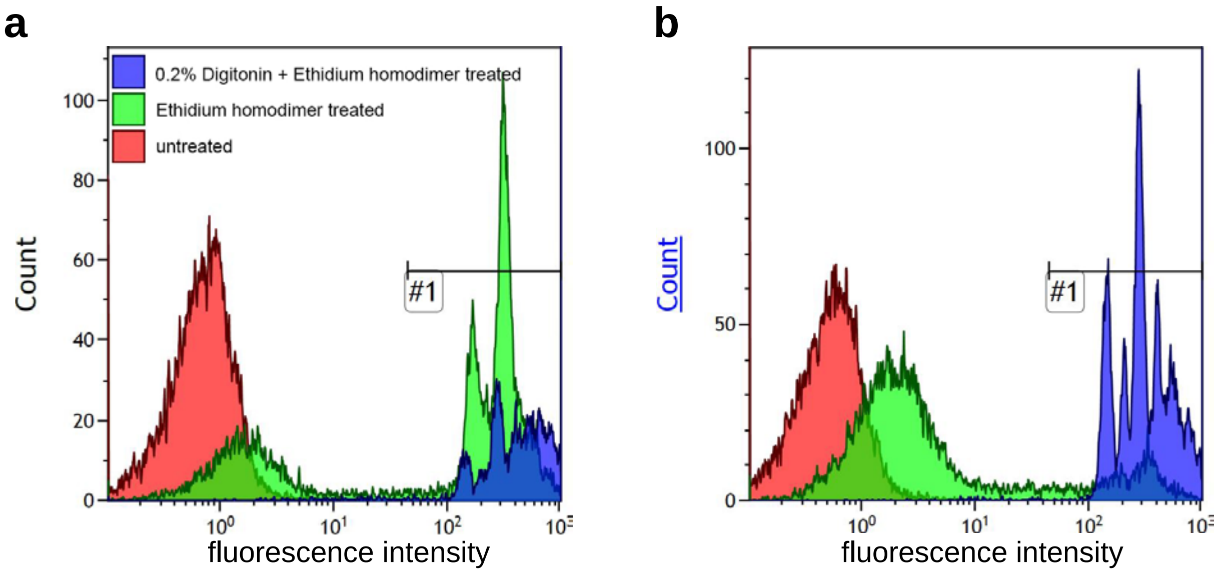

Supplementary figure 1 ¦. Flow cytometry analysis of the number of dead cells after harvesting cells from TCPS plates using PBS with 10 mM EDTA (a) or from pNIPAm coated dishes by cooling down the plates (b). Harvested cells were divided in three tubes. One tube was treated with 0.2% digitonin to permeabilize and kill the cells and after washing subsequently staining with ethidium homodimer, one tube was only treated with ethidium homodimer and one was left untreated. Of each cell the ethidium homodimer fluorescence intensity and the histogram plotted. Of all cells the ones exhibiting intensity above threshold defined by region #1 were taken as dead cells. Representative results from M1-like polarized cells of one experiment are shown.

a

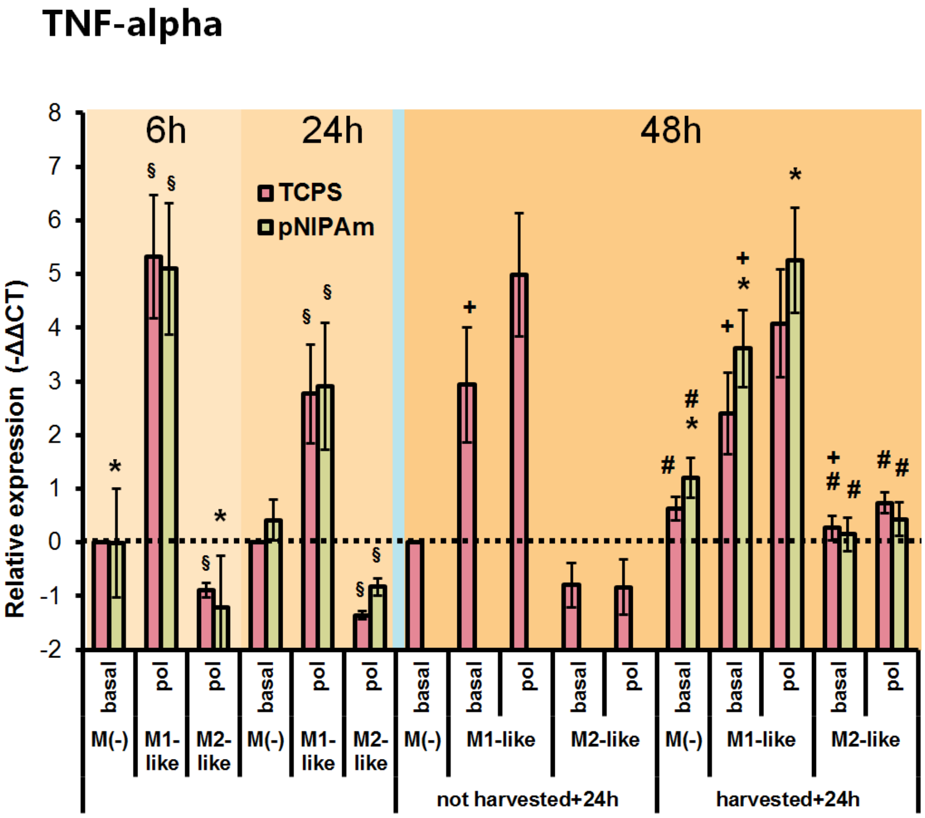
TNF-alpha
Relative expression (-ΔΔCT)
6h
24h
48h
TCPS
pNIPAm
basal
pol
M(-)
M1-like
M2-like
not harvested+24h
harvested+24h

b

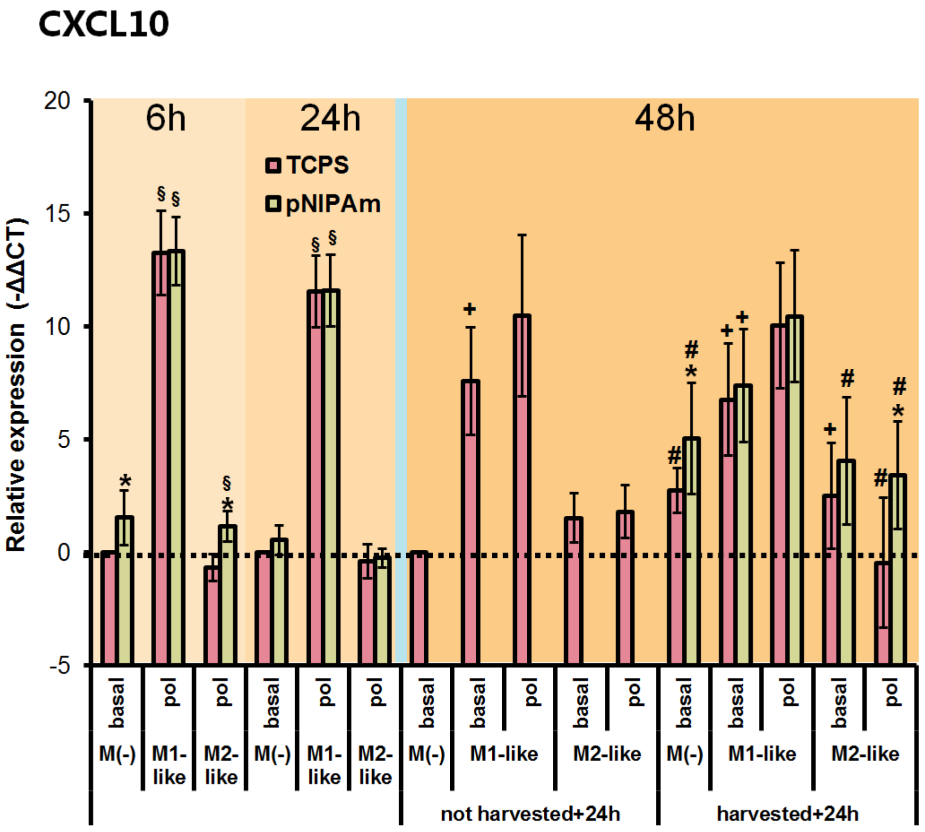
CXCL10
Relative expression (-ΔΔCT)
6h
24h
48h
TCPS
pNIPAm
basal
pol
M(-)
M1-like
M2-like
not harvested+24h
harvested+24h

c

CCL22

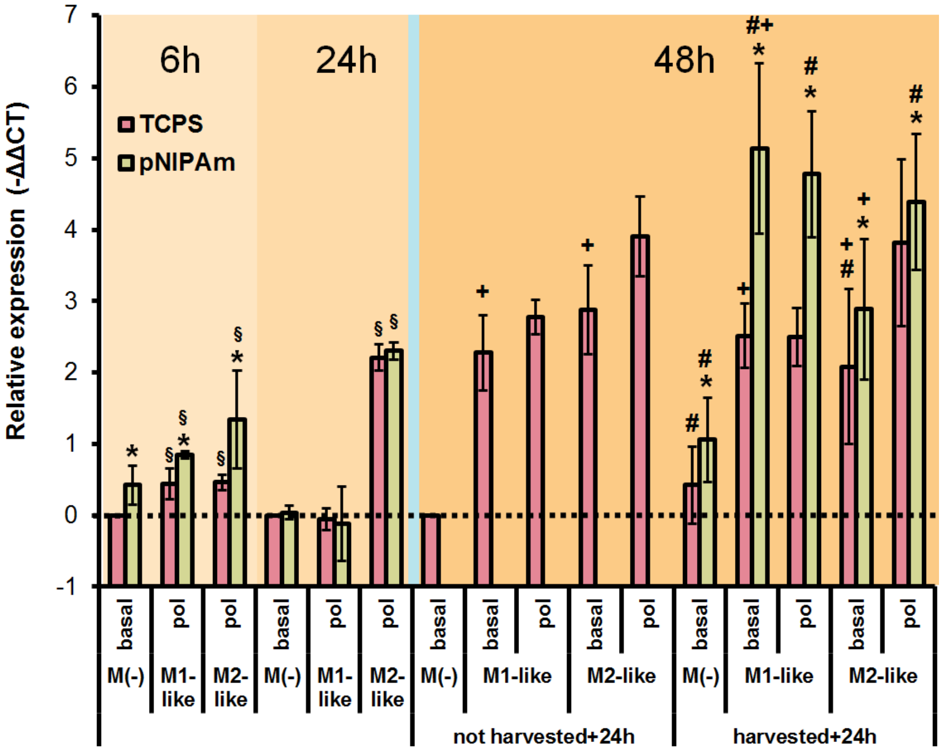

d

IL-10

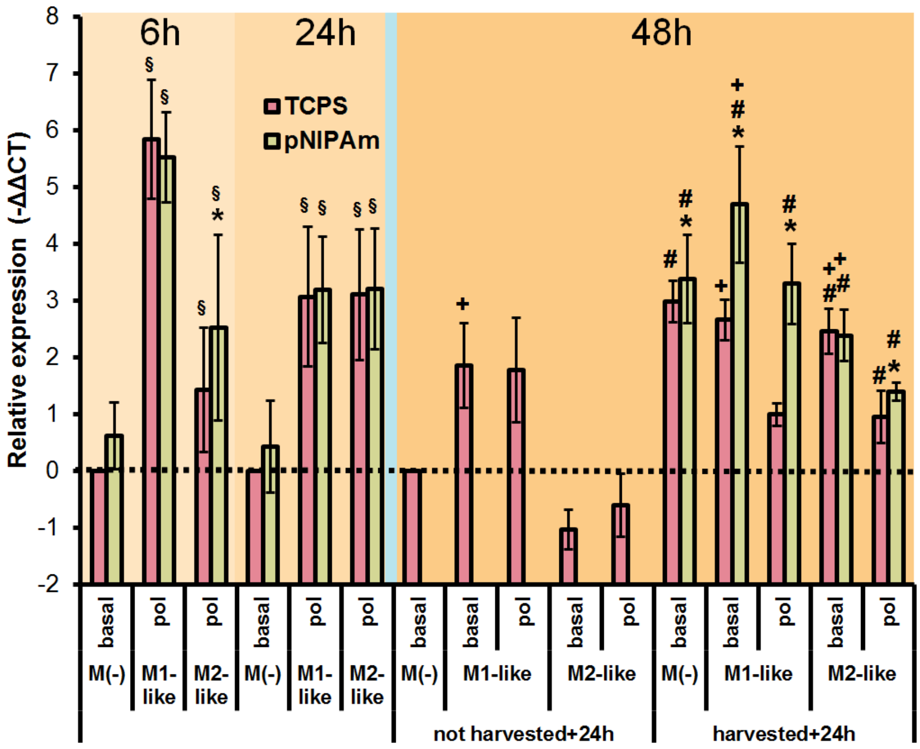

e

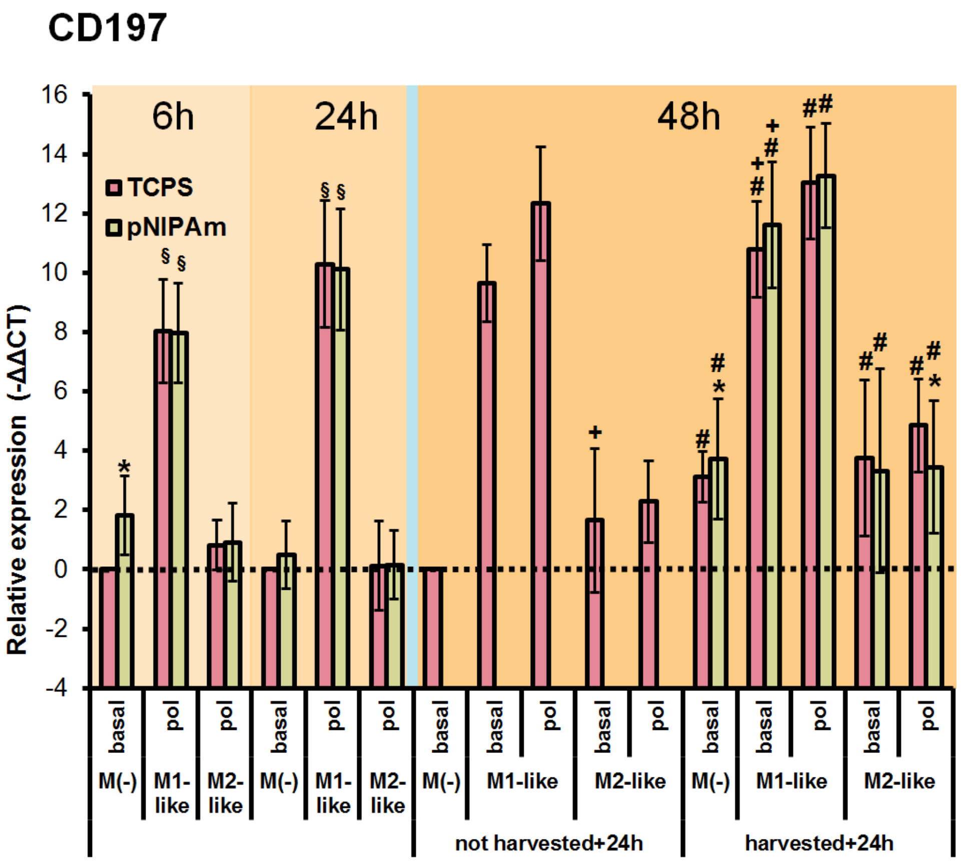
CD197
6h
24h
48h
TCPS
pNIPAm
Relative expression (-ΔΔCT)
basal
pol
M(-)
M1-like
M2-like
not harvested+24h
harvested+24h

f

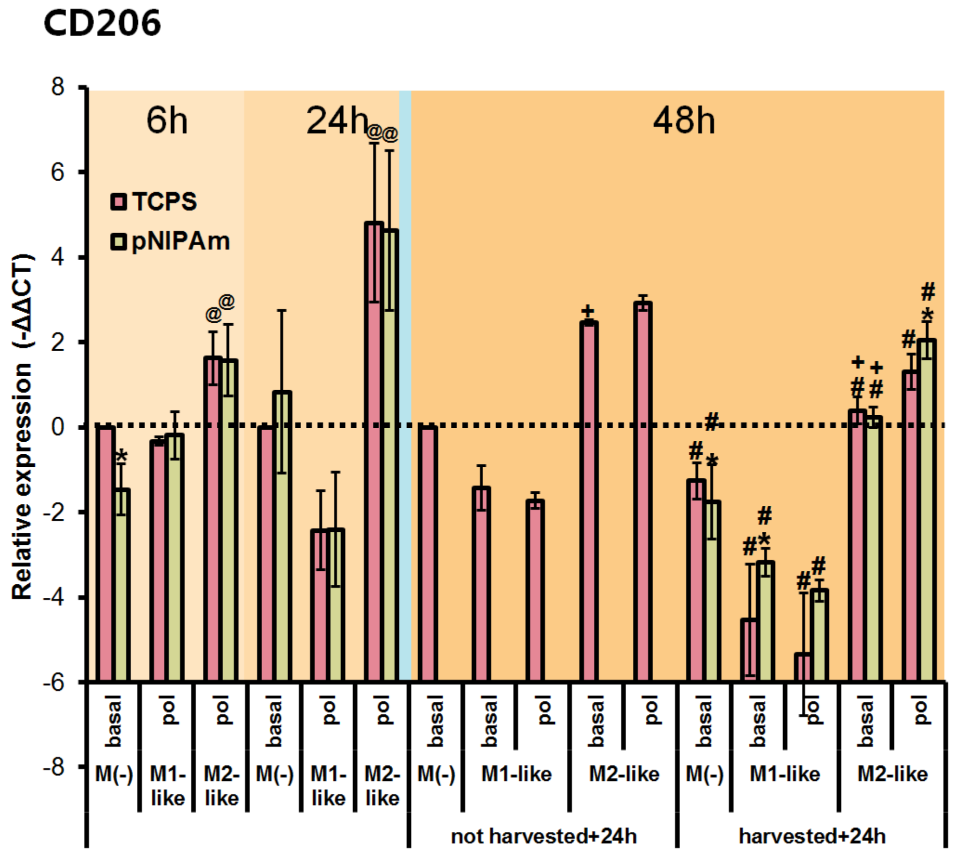
CD206
6h
24h
48h
TCPS
pNIPAm
Relative expression (-ΔΔCT)
basal
pol
M(-)
M1-like
M2-like
not harvested+24h
harvested+24h

Supplementary figure 2 ¦ Gene expression of TNF-α (A), CXCL10 (B), CCL22 (C), IL-10 (D), CD197 (D) and CD206 (F) analysed by RT-PCR at 6, 24 and 48h after start of macrophage polarization step. GAPDH was used as reference gene. The zero-level represents the -ΔΔCt of M(-) cultures on TCPS. Data of 3 independent experiments are presented as mean±sdev. *****: significantly different from identically treated cultures but cultured on TCPS surface. §: significantly different from M(-) state cultured on TCPS of the same culture time and time point. **+**: significant difference in values comparing identically treated cultures that in the period 24 to 48h after start of the polarization are kept in polarization medium (pol) instead of in basal medium (basal). **#**: significantly different from not harvested, identically treated cultures kept of TCPS ($p < 0.05$). It is interesting to note that in our study the M1- and M2-like polarized cells exhibited the M1- or M2-like gene expression related markers more prominently if harvested from pNIPAm. Furthermore, the state of differentiation (as measured by gene expression) is not as stable as generally assumed (e.g. [1,2]). By reseeding polarized cells in basal, instead of polarizing medium, the gene expression of M1-like related markers in case of M1-like cells and of M2-like related markers in case of M2-like cells was significantly reduced 24 h after reseeding. Similar observations were made when cells were not harvested and only medium was replaced. This means that if polarized cells are withdrawn from polarization stimuli, the macrophages start to depolarize. This may have significant consequences for experimental strategies in general, with effects on pre-polarized macrophages as focus.

a

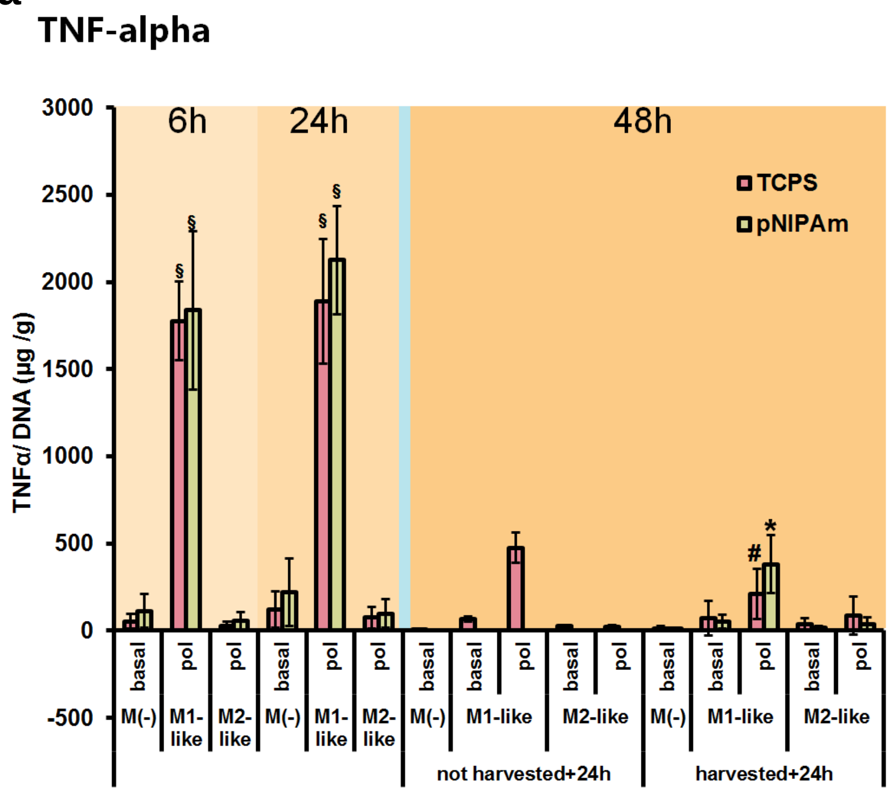

b

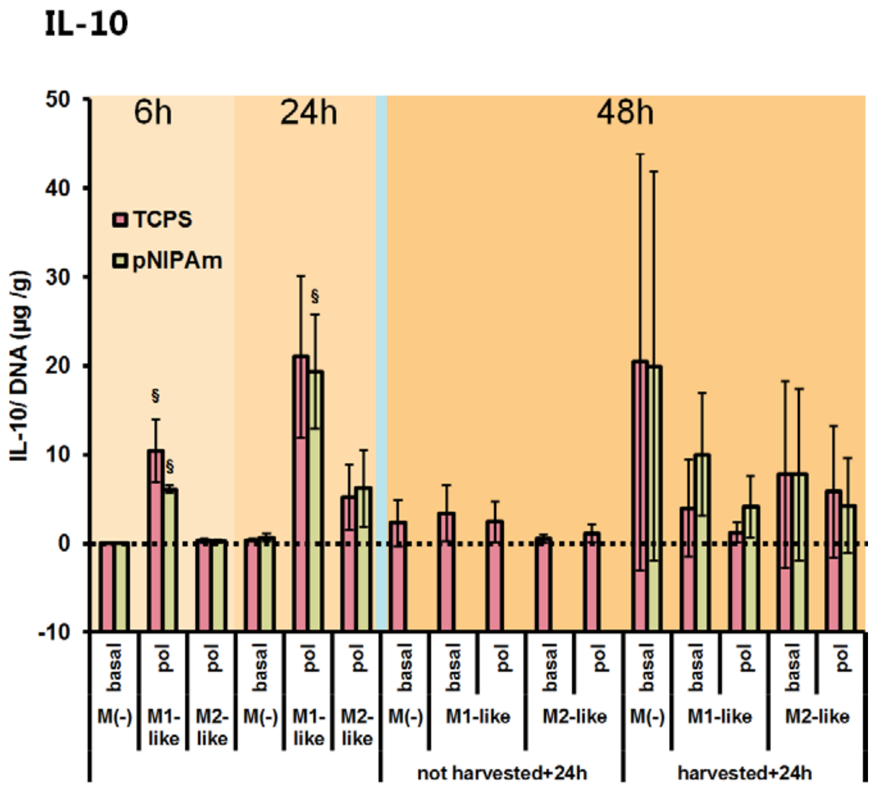

Supplementary figure 3 ¦ TNF-α (A) and IL-10 (B) protein content in medium per total DNA amount extracted from adhered not stimulated cells (M(-)) and after stimulation towards M1- and M2-like functional states at different time points. Data were collected 6h, 24h and 48h (= not harvested +24h) after stimulation and 24h after cell harvesting and re-seeding (48h = harvested +24h). The zero-value represent the control level, i.e. M(-) cultures kept in basal medium (basal) of the different time points. *****: significantly different from identically treated cultures but cultured on TCPS. **§**: significantly different from resting state THP-1 macrophages M(-) cultured on TCPS of the same culture time and time point. #: significantly different from not harvested, identically treated cultures on TCPS ($p < 0.05$).

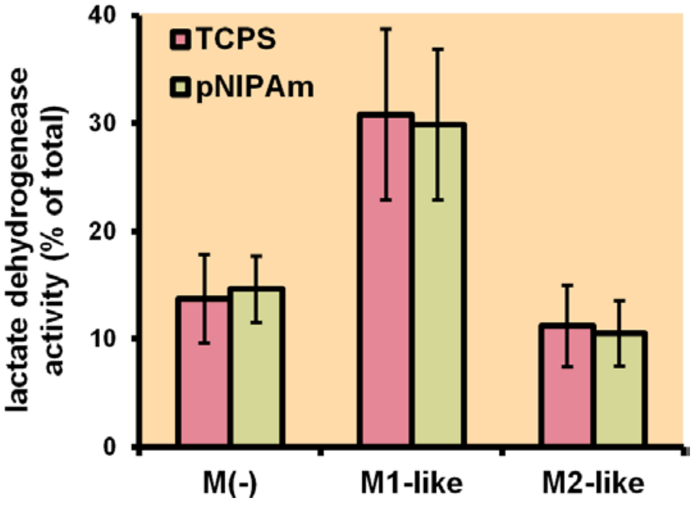

Supplementary figure 4 ¦ Lactate dehydrogenase activity in the medium before harvesting and after 24 h of stimulation with LPS (100 ng/ml) and interferon-γ (20 ng/ml) to obtain M1-like phenotype, with IL-4 (20 ng/ml) to obtain the M2-like phenotype or without additions to keep cells in the resting M(-) state. Data from 3 independent experiments are presented as mean±sdev. No significant differences were found between cultures on TCPS and pNIPAm comparing cells of the same functional state of polarization.

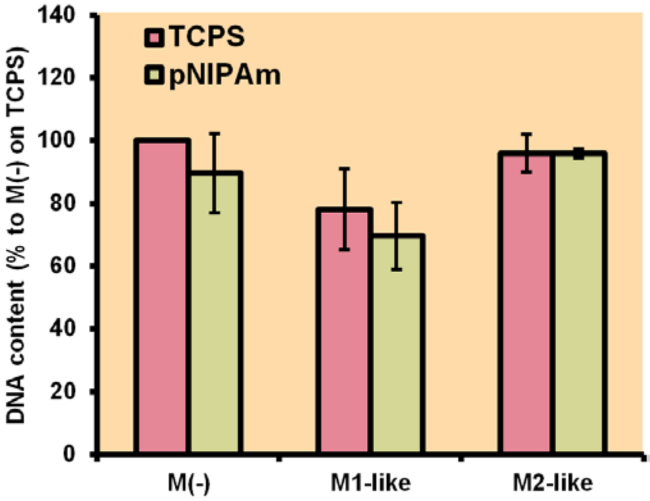

Supplementary figure 5 ¦ Total DNA content of M(-), M1 and M2a cultures on TCPS and pNIPAm plates 24 h after stimulation towards M1- and M2-like functional state and after removal of non-adherent cells. Cultures kept in basal medium are considered as resting state M(-) macrophages. Data are presented relative to M(-) cultures on TCPS as mean±sdev over 3 independent experiments. No significant differences were found between cultures on TCPS and pNIPAm comparing cells of the same functional state of polarization.

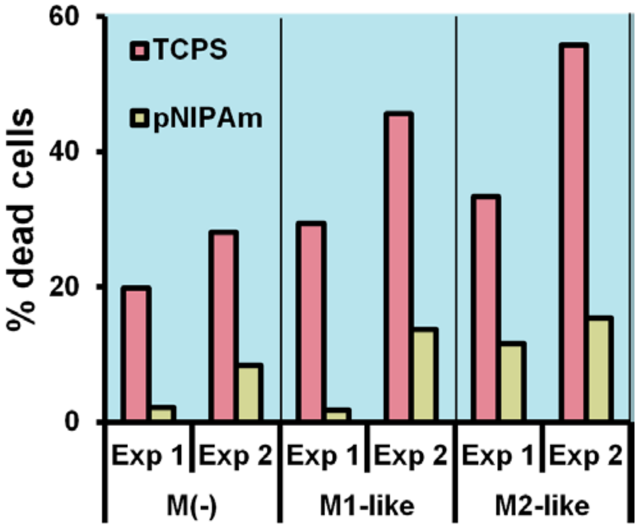

Supplementary figure 6 ¦ Percentage of dead cells in the obtained cell suspensions measured by trypan blue exclusion assay after harvesting cells from TCPS culture dishes using EDTA treatment followed by scraping (TCPS) and from pNIPAm coated culture dishes by cooling down the plates (pNIPAm). Results of two experiments are shown.

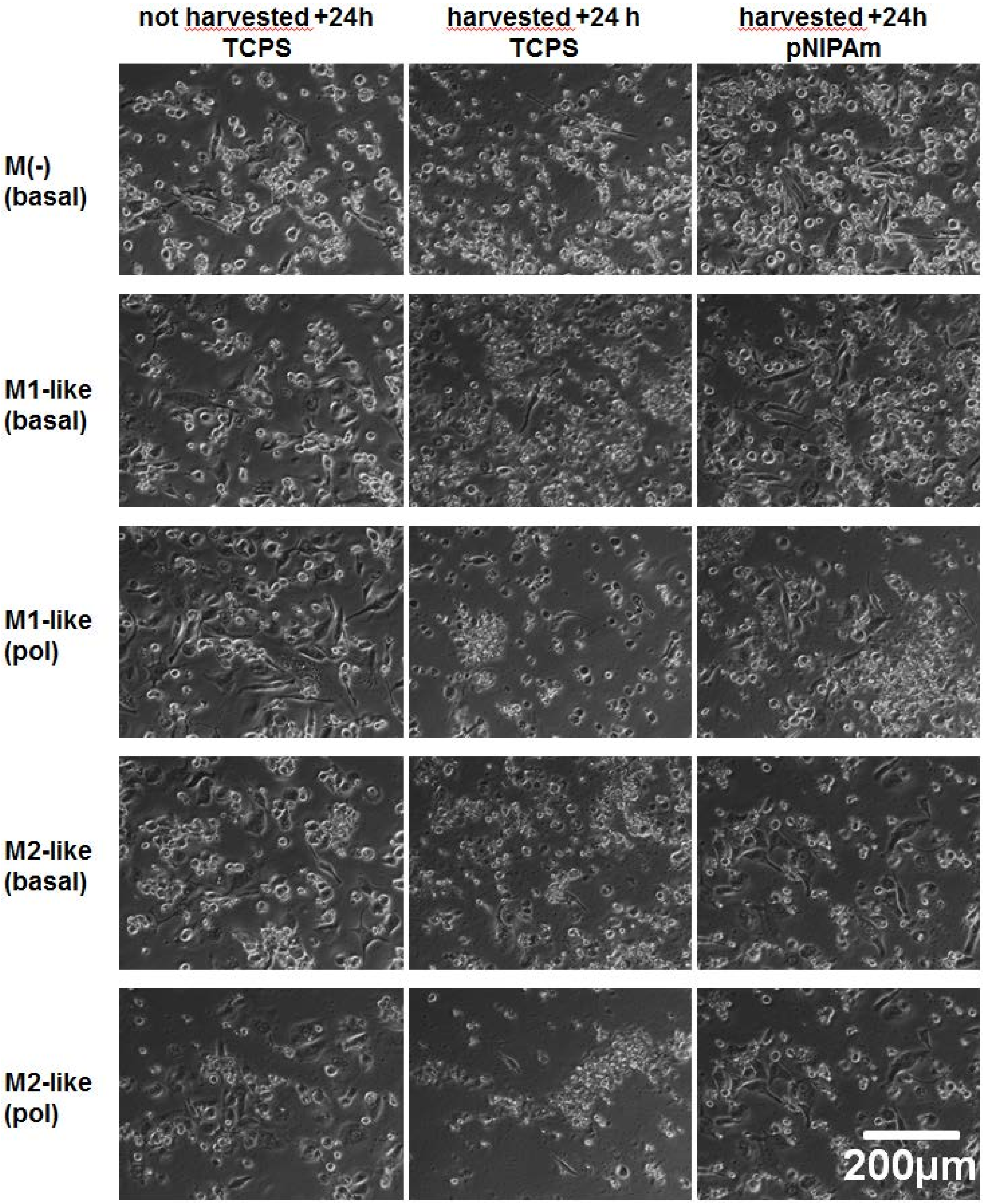

Supplementary figure 7 ¦ Light microscopy pictures of THP-1 cultures 24 h after reseeding. Cells were harvested from TCPS and pNIPAm plates and reseeded on TCPS plates in corresponding polarization medium (pol) or basal medium (basal). Pictures were taken before removal of non-adherent cells.

References

1 Saclier, M. *et al.* Differentially activated macrophages orchestrate myogenic precursor cell fate during human skeletal muscle regeneration. *Stem Cells* **31**, 384-396 (2013).

2 Nicolaidou, V. *et al.* Monocytes induce STAT3 activation in human mesenchymal stem cells to promote osteoblast formation. *PLoS One* **7**, e39871 (2012).
